# The radiomics-clinical nomogram for predicting the response to initial superselective arterial embolization in renal angiomyolipoma, a preliminary study

**DOI:** 10.3389/fonc.2024.1334706

**Published:** 2024-03-05

**Authors:** Liu Zechuan, Lyu Tianshi, Li Tiantian, Cao Shoujin, Yao Hang, Yao Ziping, Guan Haitao, Fan Zeyang, Zou Yinghua, Wang Jian

**Affiliations:** Department of Interventional and Vascular Surgery, Peking University First Hospital, Beijing, China

**Keywords:** radiomics, renal angiomyolipoma (RAML), superselective arterial embolization (SAE), response, tumor volume reduction, radiomics-clinical nomogram

## Abstract

**Purpose:**

The aim of this study was to explore a radiomics-clinical model for predicting the response to initial superselective arterial embolization (SAE) in renal angiomyolipoma (RAML).

**Materials and methods:**

A total of 78 patients with RAML were retrospectively enrolled. Clinical data were recorded and evaluated. Radiomic features were extracted from preoperative contrast-enhanced CT (CECT). Least absolute shrinkage and selection operator (LASSO) and intra- and inter-class correlation coefficients (ICCs) were used in feature selection. Logistic regression analysis was performed to develop the radiomics, clinical, and combined models where the fivefold cross-validation method was used. The predictive performance and calibration were evaluated by the receiver operating characteristic (ROC) curve and calibration curve. Decision curve analysis (DCA) was used to measure clinical usefulness.

**Results:**

The tumor shrinkage rate was 29.7% in total, and both fat and angiomyogenic components were significantly reduced. In the radiomics model, 12 significant features were selected. In the clinical model, maximum diameter (*p* = 0.001), angiomyogenic tissue ratio (*p* = 0.032), aneurysms (*p* = 0.048), and post-SAE time (*p* = 0.002) were significantly associated with greater volume reduction after SAE. Because of the severe linear dependence between radiomics signature and some clinical parameters, the combined model eventually included Rad-score, aneurysm, and post-SAE time. The radiomics-clinical model showed better discrimination (mean AUC = 0.83) than the radiomics model (mean AUC = 0.60) and the clinical model (mean AUC = 0.82). Calibration curve and DCA showed the goodness of fit and clinical usefulness of the radiomics-clinical model.

**Conclusions:**

The radiomics-clinical model incorporating radiomics features and clinical parameters can potentially predict the positive response to initial SAE in RAML and provide support for clinical treatment decisions.

## Introduction

Renal angiomyolipoma (RAML) is a mesenchymal benign kidney neoplasm that is made up of three components in varying proportions, namely, mature adipose tissue, smooth muscle, and blood vessels (1). A screening study for renal neoplasms using ultrasound in 17,941 Japanese adults revealed that the overall rate of RAML was 0.13%, with 0.22% of female and 0.1% of male patients (2). Among RAML, 80% are sporadic and 20% combine with tuberous sclerosis, an autosomal dominant disease (3). Most RAMLs are asymptomatic and are occasionally diagnosed, but they can still cause severe symptoms and complications, such as acute nontraumatic flank pain, palpable flank masses, and fulminant hypovolemic shock, which are called Wunderlich syndrome (4). Tumor size is one of the recognized reasons of symptoms and spontaneous rupture (5, 6). Therefore, it is important to control RAML growth for relieving symptoms and reducing rupture.

Historically, there was a greater trend toward surgery over embolization for renal AMLs, but the circumstance seems to change now (7, 8). Instead of nephrectomy, superselective arterial embolization (SAE) is a minimally invasive technique that can treat dysplastic vessels and reduce tumor size (9, 10). By superselecting the arteries, SAE can preserve renal function and decrease severe complications (8, 11). Because of the effectiveness and safety, SAE is also gradually used as a prophylactic treatment option to avoid RAML rupture or enlargement (12). However, there are also some patients who do not response well to SAE. Therefore, it is necessary to develop a reliable model to preoperatively predict the response to initial SAE in RAML patients.

Based on objective high-throughput imaging features, radiomics is a promising field of medical research that involves tumor segmentation, features extraction, features selection, and radiomic signature establishment (13). By extracting and evaluating features from digital images, radiomics could detect subtle changes and heterogeneity beyond human vision and converts medical images to quantitative, minable, high-dimensional data (14). To date, a model established by radiomics combined with clinical features has been widely applied in response evaluation and prognosis prediction across the field of oncology and presents great efficiency (15, 16).

Therefore, the aim of this study is to establish a radiomics-clinical model of RAML to provide the clinician with a quantitative tool for preoperatively predicting individual response to SAE.

## Materials and methods

### Patients

This retrospective study was approved by the ethics review board. The ethics committee approved that this retrospective study could waive informed consent. This study analyzed 328 patients with RAML whose data were collected from October 2017 to October 2022 from the Picture Archiving and Communication System (PACS) of the hospital. Inclusion criteria were as follows: (1) lesions that had definitive radiological diagnosis of classical RAML by contrast-enhanced computed tomography (CECT), (2) patients who underwent CECT less than 1 month before SAE, (3) patients with complete clinical-radiological data, and (4) lesions that accepted SAE for the first time. The exclusion criteria were as follows: (1) patients who underwent mTOR inhibitor, such as Everolimus, (2) patients who underwent emergency SAE due to RAML rupture, and (3) patients lost to follow-up. The patients were requested to follow up at 1, 3, 6, and 12 months in the first year after SAE and subsequently once a year.

**Figure 1:**
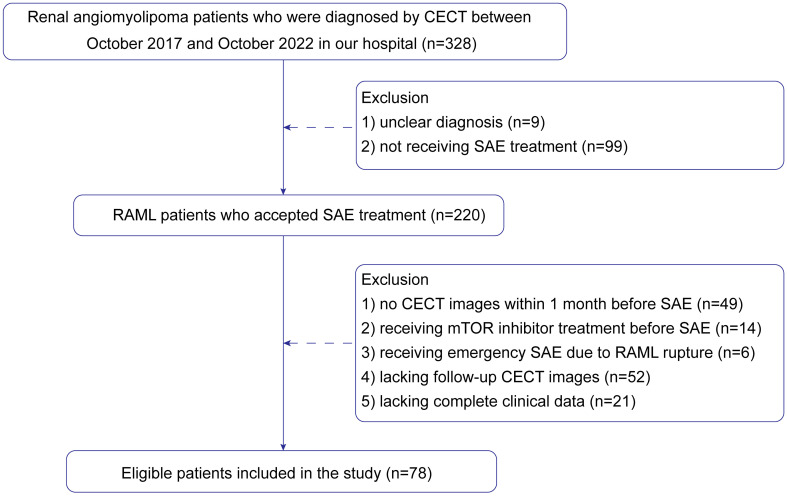
Flow chart showing the patient selection and exclusion criteria.

Finally, 78 patients were enrolled in the study. The patient recruitment process is presented in **Figure 1**. According to meta-analysis (17), the average RAML shrinkage rate was 30% after SAE. Combined with the result of this study, the lesion set was divided into a positive response group with volume reduction larger than 30% and a negative response group with volume reduction less than 30%. Baseline characteristics were collected.

### CT examination protocols

A total of 78 recruited patients took non-enhanced and CECT examinations by two commercial CT scanners. Scanner 1: Smatom Definition AS, Siemens Healthcare. The scanning parameters were as follows: tube voltage, 120 kV; tube current, 200 mA; collimation, 64 × 0.625 mm; pitch, 1.2; rotation time, 0.75 s; slice thickness, 5 mm; reconstruction thickness, 1 mm. Scanner 2: Discovery CT750 HD, GE Healthcare. The scanning parameters were as follows: tube voltage, 120 kV; tube current, 250 mA; collimation, 64 × 0.625 mm; pitch, 1.375; rotation time, 0.5 s; slice thickness, 5 mm; reconstruction thickness, 1 mm. CECT was performed based on the technology of computer-assisted bolus tracking, with nonionic contrast medium (Iopromide, Ultravist 370; Bayer Schering Pharma) being administrated into the antecubital vein via a power injector at a rate of 2.5 mL/s (1.5 mL/kg). With a 100-HU threshold in the abdominal aorta at the level of celiac artery as the baseline, the post-contrast CT of corticomedullary phase (CMP, 30 s) and nephrographic phase (NP, 90 s) was acquired. The CT images were blindly analyzed by two radiologists (reader 1, Z.L.; reader 2, T.L.) with 8 and 13 years of abdominal imaging experience, respectively.

### Selective arterial embolization

With modified Seldinger’s technique, the procedure was performed under local anesthesia through the right common femoral artery. Abdominal aortogram was accomplished using the 5F PIG angiographic catheter (Cook Medical Technologies) to relocate the renal artery and determine if there were accessory renal arteries or extra-renal feeding vessels. Then, renal arteriography was performed using the Cobra angiographic catheter (Cook Medical Technologies) to assess the tumor stain, feeders, arteriovenous fistula, and aneurysms. Afterward, RAML feeding vessels were superselected and embolized one by one with coaxial microcatheters (Asahi, Boston Scientific Corporation). For embolization, various embolic materials were used and could simply be divided into two groups: one group consisted of traditional particles and liquid embolic agents, which included lipiodol, gelatin sponge, and polyvinyl alcohol particles, while the other group was composed of drug-eluting beads (CalliSphere, 100–300 μm/300–500 μm/500–700 μm, Jiangsu Hengrui Medicine Corporation) loaded with bleomycin. The endpoint of embolization was the forward blood flow stasis in feeding arteries. Technical success was defined as complete obliteration of tumor vessels and stain according to the post-SAE angiography.

### Image processing and tumor segmentation

The thin-layer images were uploaded to 3D Slicer (version 4.10.2, https://www.slicer.org/). In order to eliminate the influence of different scanners and layer thicknesses on the radiomic features, images received preprocessing and standardization before region of interest (ROI) segmentation. First, linear interpolation was reconstructed into 1.0 mm × 1.0 mm × 1.0 mm at X/Y/Z spacing. Second, the window level and width were set to 60 HU and 360 HU. Three-dimensional (3D) segmentation of the ROI was performed using the ITK-SNAP software (version3.8.0, www.itksnap.org). The manual defined smooth curve was delineated along the borders of tumor on NP images before and after SAE, but avoided covering renal parenchyma, perirenal fatty tissues, and blood vessels. The purpose of outlining the postoperative ROI was to calculate tumor shrinkage rate and the change rate of fat or angiomyogenic components accurately. An example of the manual segmentation is presented in **Supplementary Figure 1**.

Intra- and inter-class correlation coefficients (ICCs) were used to evaluate the intra- and inter-observer reproducibility of tumor segmentation and radiomics feature extraction. Tumor segmentation was performed by radiologist 1 and radiologist 2. Radiologist 1 performed tumor segmentation on all patients and radiologist 2 randomly selected 20 patients for independent segmentation to assess inter-class agreement. Radiologist 1 repeated the same work of the previous 20 patients 1 month later to assess intra-class agreement.

### Radiomics feature extraction and model establishment

Feature extraction was performed using the PyRadiomics library (18). To prevent overfitting and selection bias, ICCs and least absolute shrinkage and selection operator (LASSO) regression were adopted to screen out the most prominent radiomic features. We evaluated the ICCs of radiomic features and selected features with an ICC of greater than 0.75. LASSO was used as a feature selector based on its sparse properties when setting the large coefficient *λ* of the L1 norm penalty, and the minimum deviation rule was employed to select the optimal coefficient *λ*. Based on the selected features, the radiomics model was established by logistic regression, support vector machine (SVM), and decision tree (DT), respectively. The radiomics score (Rad-score) was calculated for each patient via a linear equation of selected features weighted by their respective coefficients.

### Clinical model and radiomics-clinical model establishment

The independent clinical factors related to SAE efficacy were determined by univariate and multivariate logistic regression. Firstly, the clinical factors were brought into univariate logistic regression. Then, the variables with a *p*-value of less than 0.1 were further assessed using multivariate logistic regression. The clinical model was established by the independent factors.

Fat ratio within the tumor was calculated with in-house software written by Python. According to previous articles (9, 11), fat tissue was defined as the region lower than −20 HU, while the remaining tissue was specified as the angiomyogenic tissue. Tumor shrinkage rate was also calculated by Python in the basis of tumor segmentation before and after SAE. The code of the in-house software written by Python is presented in **Supplementary Table 1**.

After multicollinearity test, the radiomics-clinical model was constructed by incorporating the Rad-score and the independent clinical factors. Meanwhile, the radiomics-clinical model was visualized as a nomogram.

### Evaluation of model performance

The performance evaluation in different models contained discrimination, calibration, and clinical usefulness. Since the case set was relatively small, wide fivefold cross-validation was used in this study. Receiver operating characteristic (ROC) curves were built for each model to evaluate the discrimination performance, and the DeLong test was used to check the differences between AUCs. The calibration curve was used to assess the calibration of the nomogram. Decision curve analysis (DCA) was performed to estimate the clinical usefulness based on the net benefit at a range of threshold probabilities.

### Statistical analysis

Statistical tests were performed on Stata/SE (version15.0) and R statistical software (version 4.1.3, https://www.r-project.org). Univariate analysis and multivariate logistic regression were used to select independent clinical factors and establish different models. The DeLong test was used to distinguish differences in ROCs. The development of nomogram and calibration was performed using the “rms” package. The DCA was performed using the “dcurves” package. *p*-value < 0.05 (two-sided) was considered as statistical significance.

## Results

### Patients

All RAMLs were definitively diagnosed by the presence of macroscopic fat tissue in CT. A total of 78 RAML patients, namely, 39 positive response patients and 39 negative response patients, were enrolled in this study. Baseline characteristics are summarized in **Table 1**.

**Table 1:** Baseline clinical characteristics.

Characteristic	Data
Gender (*n*, %)	
Male Female	9 (11.5%) 69 (88.5%)
Age (years, mean ± SD)	41.7 ± 13.9
BMI	23.2 ± 3.2
Hypertension (*n*, %)	
Yes No	11 (14.1%) 67 (85.9%)
Diabetes (*n*, %)	
Yes No	6 (7.7%) 72 (92.3%)
TSC (*n*, %)	
Yes No	19 (24.4%) 59 (75.6%)
Symptoms (*n*, %) *	
Present Absent	52 (66.7%) 26 (33.3%)
Hematuria (*n*, %)	
Present Absent	24 (30.8%) 54 (69.2%)
Pregnancy (*n*, %)	
Yes No	4 (5.1%) 74 (94.9%)
Number (*n*, %)	
Single Multiple	34 (43.6%) 44 (56.4%)
Aneurysms (*n*, %)	
Present Absent	57 (73.1%) 21 (26.9%)
Maximum diameter (cm, mean ± SD)	9.2 ± 5.3
Tumor volume (cm^3^, median, range)	132.8 (24.9–2,044.1)
Fat ratio (%, mean ± SD)	45.9 ± 28.2
Angiomyogenic tissue ratio (%, mean ± SD)	59.8 ± 27.6
Mean CT value (HU, median, range)	−3.1 (-66.3–115.5)
Creatine (μmol/L, mean ± SD)	69.8 ± 21.7
WBC (*10^9^, mean ± SD)	5.8 ± 2.7

TSC, tuberous sclerosis complex; SD, standard deviation; WBC, white blood cell.

*Symptoms, including pain, shock, or retroperitoneal bleeding.

### Selective arterial embolization

The characteristics and outcomes of SAE are listed in **Table 2**. Both maximum diameter and volume of tumors were reduced after SAE with statistical differences. Tumor shrinkage rate was 29.7% in total. Both angiomyogenic tissue and fat tissue shrank significantly, with angiomyogenic components shrinking better. The creatine level was slightly elevated after SAE without statistical significance (69.8 ± 21.7 vs. 74.5 ± 22.4 μmol/L, *p* = 0.187). The post-embolization syndrome (PES) occurred in 59% of patients, which was consistent with previous studies (10, 17). There were no severe complications in this study. SAE was technically successful for 75 of the 78 RAMLs (**Supplementary Figure 2**). Two RAMLs were combined with TSC and the lesions were widely distributed in bilateral kidneys, so only moderate devascularization was achieved to protect the renal function and reduced complications after SAE. Another RAML had the partial region with multiple small feeding arteries, which were impossible to superselective catheterize.

**Table 2:** Clinical variables post-SAE.

Variables	Result	*p*-value
Maximum diameter (cm, mean ± SD)		<0.0001
Pre Post	9.2 ± 5.3 7.3 ± 4.1	
Tumor volume (cm^3^, median, range)		0.0001
Pre Post	132.8 (24.9–5,795.2) 93.1 (9.0–5,637.3)	
Tumor shrinkage rate (%, median, range)	29.7 (−34.6–88.2)	
Fat ratio (%, mean ± SD)		<0.0001
Pre Post	45.9 ± 28.2 36.9 ± 27.8	
Angiomyogenic tissue ratio (%, mean ± SD)		<0.0001
Pre Post	59.8 ± 27.6 40.8 ± 26.2	
Creatine (μmol/L, mean ± SD)		0.187
Pre Post	69.8 ± 21.7 74.5 ± 22.4	
Embolic material (*n*, %)		
Lipiodol and particles	64 (82.1%)	
CalliSphere + bleomycin	14 (17.9%)	
Technical success rate (*n*, %)	75 (96.2%)	
Post-embolization syndrome (PES, *n*, %)		
Present Absent	46 (59.0%) 32 (41.0%)	
Post-SAE time (months, median, range) *	4.35 (1–25.97)	

SAE, superselective arterial embolization; SD, standard deviation.

*From SAE to the latest follow-up.

### Features extraction, selection, and radiomics signature establishment

A total of 106 radiomics features were extracted including first order, geometry, and texture features, which were defined based on the following matrices: gray-level cooccurrence matrix (GLCM), gray-level run length matrix (GLRLM), gray-level size zone matrix (GLSZM), neighboring gray tone difference matrix (NGTDM), and gray-level dependence matrix (GLDM).

**Figure 2:**
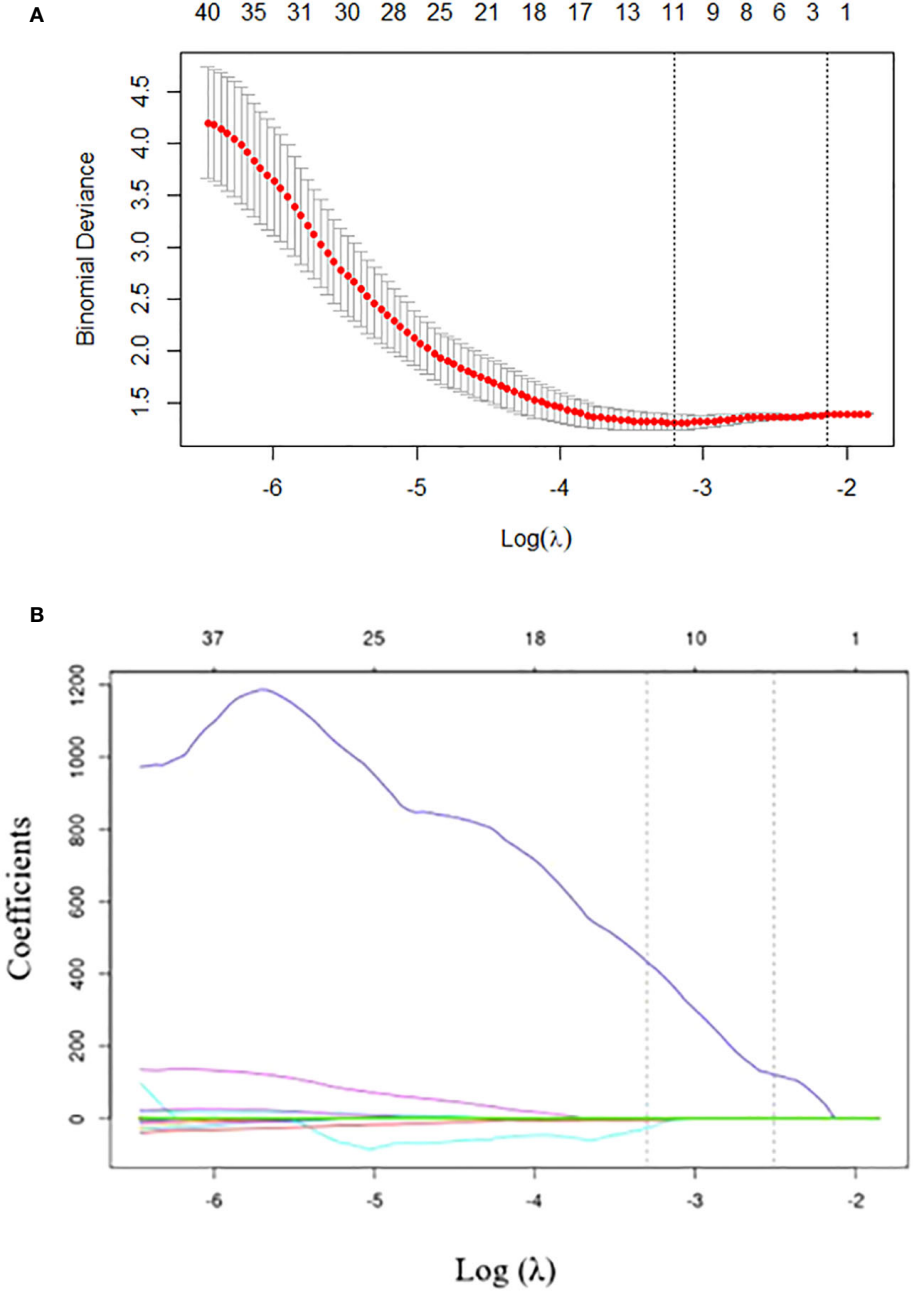
Radiomics feature selection using the least absolute shrinkage and selection operator (LASSO) regression. **(A)** Tuning parameter (λ) selection in LASSO model performed by 10-fold cross-validation via minimum criteria. The optimal value of the LASSO tuning parameter (λ) is indicated by the dotted vertical line on the left, with a λ value of 0.037. The dotted line on the right denotes 1−standard error criterion (1−SE). **(B)** LASSO coefficient profiled of the 80 texture features. The left vertical line is drawn at the optimal log(λ) value selected using 10-fold cross-validation, which results in 12 selected radiomics features.

To eliminate redundancy, radiomics features with an ICC of less than 0.75 were excluded and 80 features were retained. The 80 features were reduced to 12 by LASSO regression with the optimal coefficient λ = 0.037 (**Figure 2**). Based on the selected features, the radiomics model was established by logistic regression, SVM, and DT with AUCs of 0.597, 0.650, and 0.504, respectively. The selected radiomic features are presented in **Supplementary Table 2**.

### Clinical model establishment

The clinical characteristics submitted to univariate analysis are shown in **Table 3**. Clinical factors with *p*<0.1 were introduced in the next analysis. In the process, we found that there were collinearity problems between fat ratio and angiomyogenic tissue ratio. According to clinical experience and previous studies (17, 19), angiomyogenic tissue ratio remained. No multicollinearity was found among other independent variables.

**Table 3:** Univariate and multivariate logistic regression analysis.

Variables	Univariable analysis		Multivariable analysis	
	*p*-value	Odds ratio (95% CI)	*p*-value	Odds ratio (95% CI)
Gender	0.095	0.247 (0.479–1.275)	0.342	0.345 (0.038-3.101)
Age	0.921	0.998 (0.967–1.301)	–	–
Hypertension	0.745	1.236 (0.344–4.446)	–	–
Diabetes	1.000	1.000 (0.189–5.289)	–	–
TSC	0.792	0.870 (0.309–2.449)	–	–
Symptoms	0.152	0.496 (0.189–1.296)	–	–
Hematuria	0.328	1.624 (0.614–4.292)	–	–
Pregnancy	0.328	0.315 (0.031–3.177)	–	–
Number	0.362	1.520 (0.617–3.739)	–	–
Aneurysm	0.079	2.560 (0.898–7.295)	0.048	5.413 (1.016–28.839)
Maximum diameter	0.015	0.869 (0.776–0.972)	0.001	0.729 (0.601–0.885)
Tumor volume *	0.139	0.999 (0.999–1.000)	–	–
Fat ratio	0.006	0.878 (0.0152–0.504)	–	–
Angiomyogenic tissue ratio	0.005	13.460 (2.201–82.285)	0.032	2,279 (1.978–2,626,660)
Mean CT value	0.008	1.017 (1.004–1.030)	0.097	0.959 (0.913–1.008)
WBC *	0.97	1.096 (0.910–1.319)	–	–
Creatine *	0.060	1.040 (0.998–1.083)	0.450	1.011 (0.963–1.062)
Embolic agent **	0.556	1.419 (0.442–4.557)	–	–
PES	1.00	1.000 (0.406–2.465)	–	–
Post-SAE time	0.026	1.124 (1.014–1.247)	0.002	1.364 (1.124–1.655)

TSC, tuberous sclerosis complex; PES, post-embolization syndrome.

Fat ratio was excluded from multivariable analysis because of the collinearity problem with angiomyogenic tissue ratio.

*Tumor characteristic or test results before SAE.

** Lipiodol + particles or CalliSphere + bleomycin.

**Figure 3:**
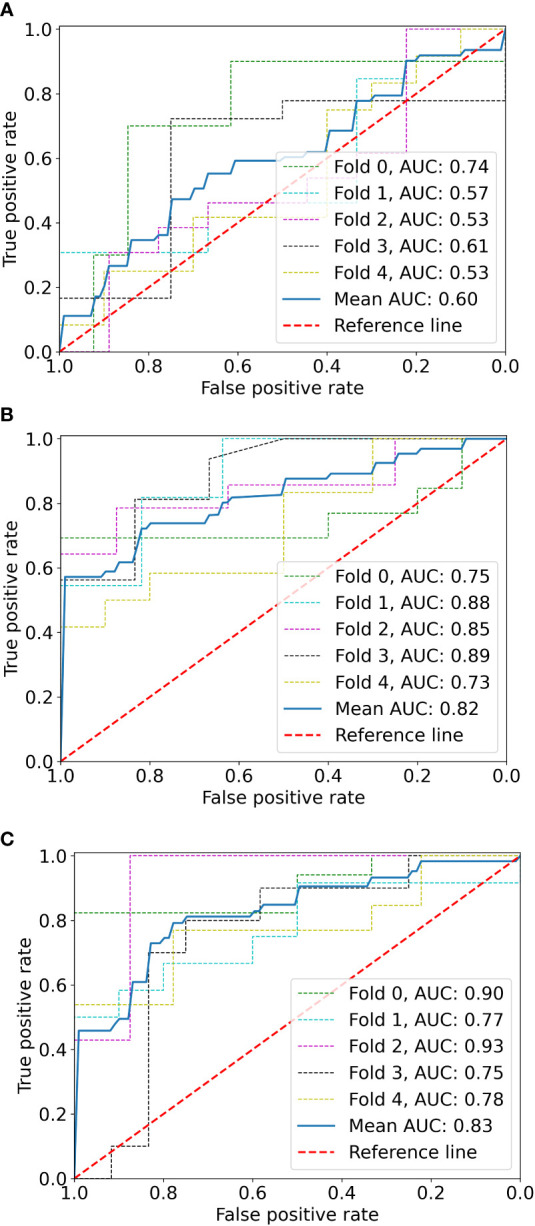
The ROC curves of the radiomics model **(A)**, clinical model **(B)**, and radiomics-clinical model **(C)** based on fivefold cross-validation. The mean AUC of the radiomics-clinical model is outperformed compared to radiomics the model and clinical model.

The logistic stepwise regression model was used to ensure the independent factors of positive response to SAE (**Table 3**). RAMLs with a smaller maximum diameter (OR = 0.729, *p* = 0.001), a higher angiomyogenic tissue ratio (OR = 2,279, *p* = 0.032), aneurysms (OR = 5.413 *p* = 0.048), and a longer post-SAE time (OR = 1.364, *p* = 0.002) might have a positive response to SAE. The clinical model was established and further validated by a fivefold test. The ROC curve is shown in **Figure 3B** (mean AUC = 0.822).

### Radiomics-clinical model establishment and evaluation

**Figure 4:**
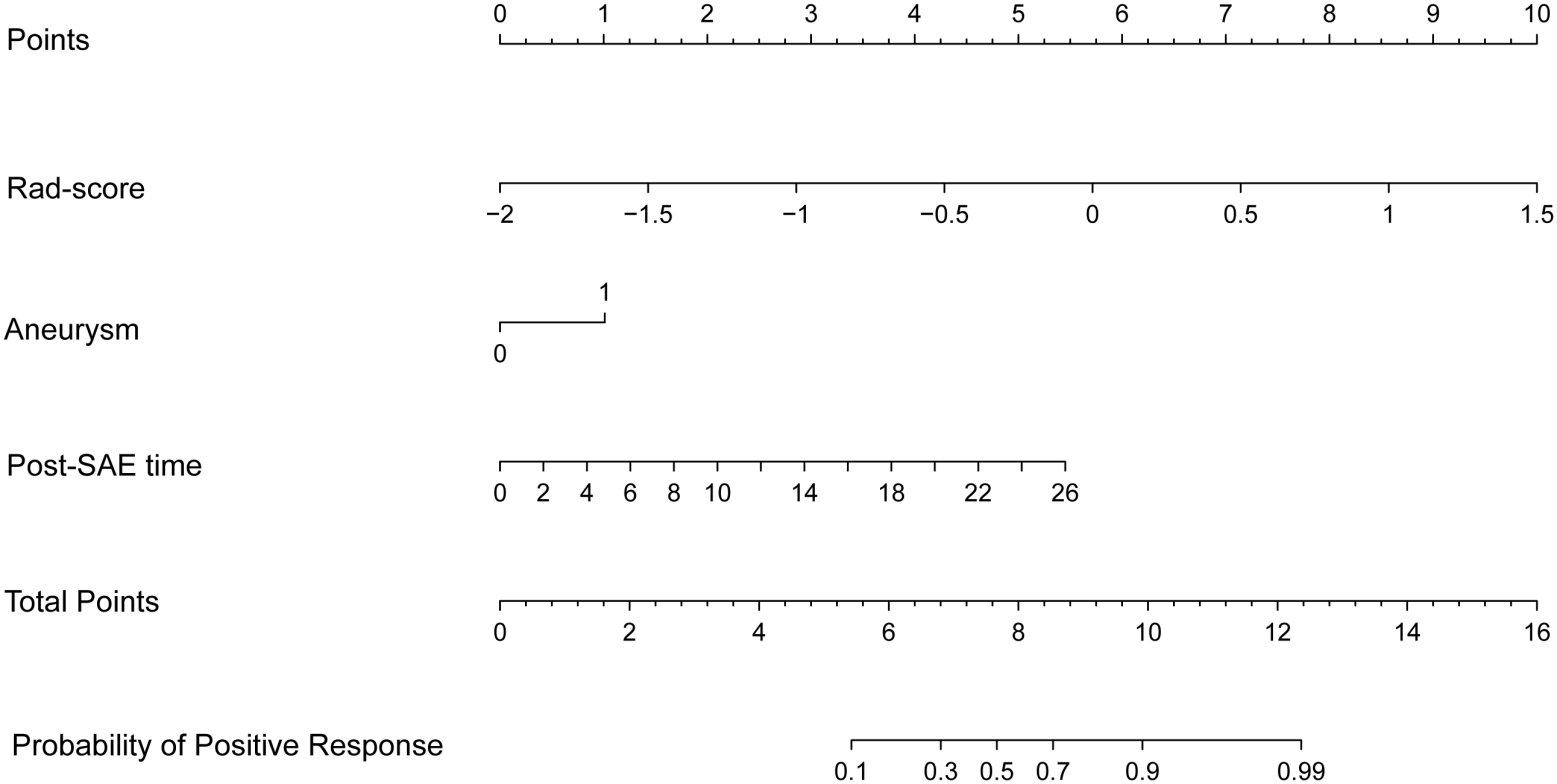
The radiomics-clinical nomogram. Combining Rad-score, aneurysm, and post-SAE time, the radiomics-clinical nomogram can predict the probability of the positive response to SAE individually.

Before combining the Rad-score and clinical factors, the reliability of features was assessed by the concordance correlation coefficient to avoid the underlying severe linear dependence. Features with a Spearman correlation coefficient of more than 0.6 were removed from further analysis (**Supplementary Table 3**). In the test, angiomyogenic tissue ratio and maximum diameter showed extremely strong correlations with the Rad-score, which suggested that certain tumor information might be involved in the radiomics signature. A combined radiomics-clinical model, which was presented in the form of a nomogram (**Figure 4**), was established by incorporating Rad-score, aneurysm, and post-SAE time. The fivefold cross-validated ROC curves are displayed in **Figure 3C** and the mean AUC was 0.834.

The ROCs of each model are presented in **Figure 3**, and the diagnostic performance is shown in **Table 4**. The DeLong test showed that the AUCs had no statistically significant differences between the models (**Supplementary Table 4**). The Nomo-score was calculated using the following formula: Nomo-score = 2.796 × Rad-score + 0.989 × aneurysm + 0.205 × post-SAE time − 1.911.

**Table 4:** Diagnostic performance of different models.

	SEN	SPE	ACC	AUC (95% CI)
Radiomics model				
Logistic	0.67	0.55	0.61	0.597 (0.530–0.726)
DT	0.51	0.48	0.50	0.504 (0.333–0.675)
SVM	0.66	0.50	0.58	0.650 (0.480–0.783)
Clinical model	0.74	0.81	0.77	0.822 (0.735–0.890)
Radiomics-clinical model	0.79	0.78	0.77	0.834 (0.762–0.927)

Logistic, logistic regression; DT, decision tree; SVM, support vector machine; SEN, sensitivity; SPE, specificity; ACC, accuracy; AUC, area under the curve; CI, confidence interval.

**Figure 5:**
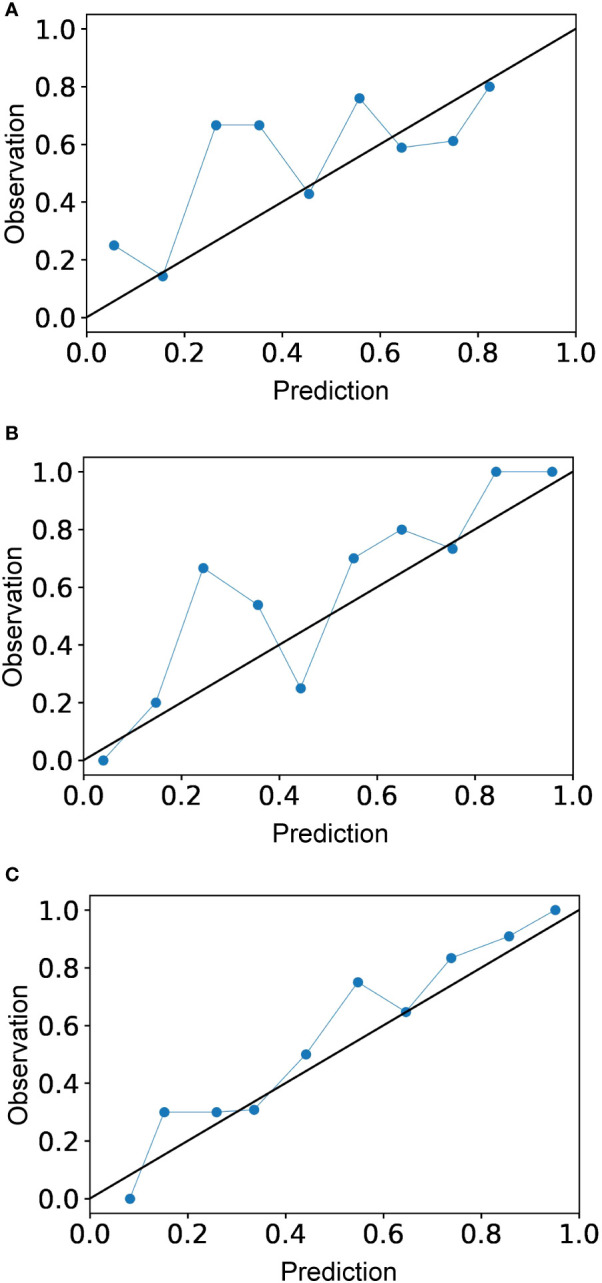
Calibration curves for the radiomics model **(A)**, clinical model **(B)**, and radiomics-clinical model **(C)**. Calibration curve indicates the goodness of fit of the radiomics-clinical model, which is better than other models.

**Figure 6:**
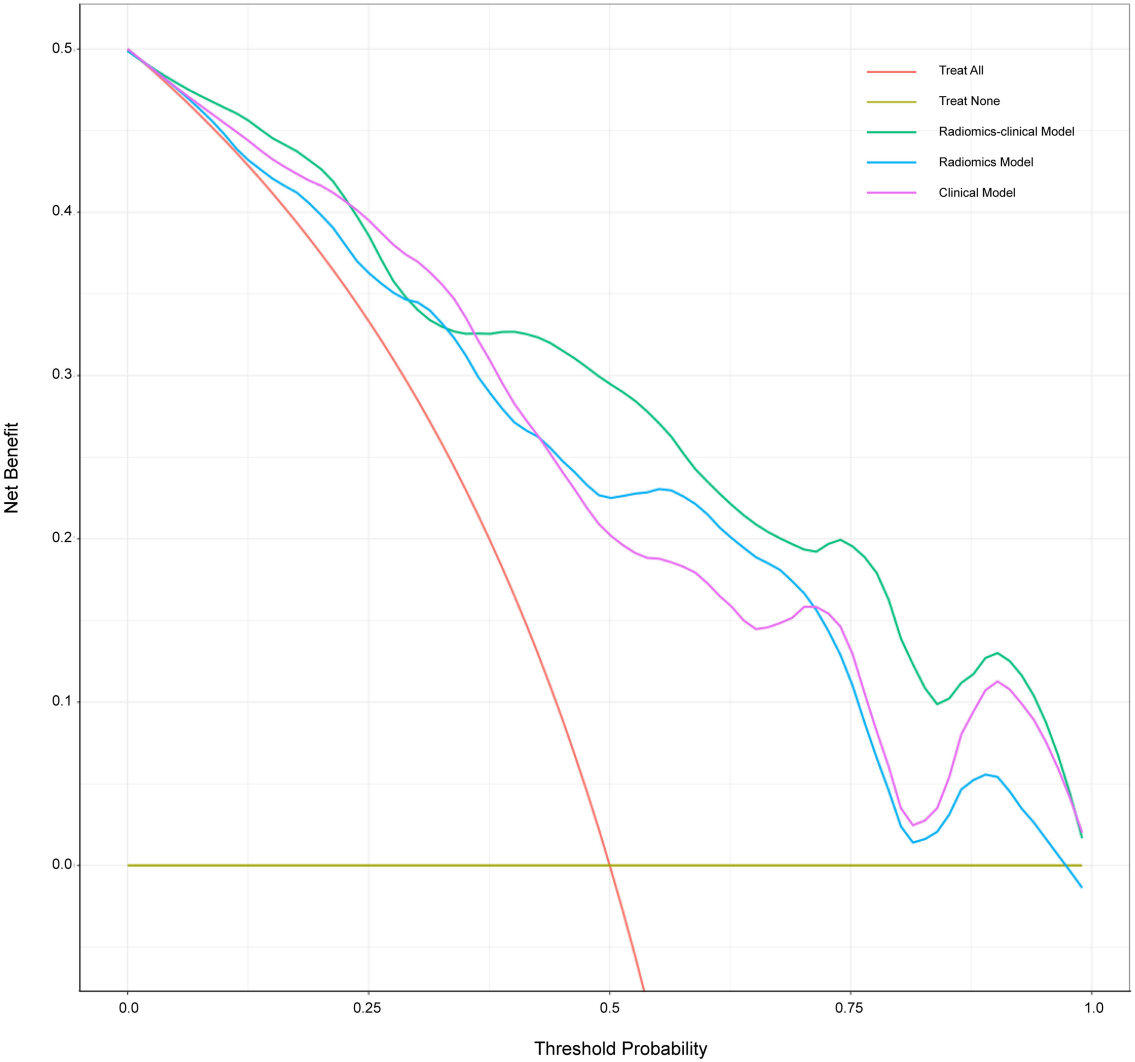
Decision curve analysis for three models. The *x*-axis indicates threshold probability; the *y*-axis represents the net benefit. The rad line represents the assumption that all patients have a positive response to SAE. However, the yellow line is the opposite. The green line, purple line, and blue line represent net benefit of the radiomics-clinical model, the clinical model, and the radiomics model, respectively. The radiomics-clinical model has a higher net benefit compared with the other models at the range of threshold probability.

The calibration curve is shown in **Figure 5**. The calibration prediction curve fitted well with the ideal curve in the radiomics-clinical model, indicating the good fit of the nomogram. The DCA (**Figure 6**) showed that the radiomics-clinical model had better overall net benefits than other models at the highest range of threshold probability.

## Discussion

In this study, we successfully develop a combined radiomics-clinical model based on CECT, which shows a good performance in predicting the efficacy of initial SAE in RAML. It concludes that the Rad-score, a quantitative parameter, could serve as an independent predictor of SAE efficacy and the radiomics model combined with clinical features can improve the predictive ability. This novel approach is expected to forecast SAE outcome intuitively and support decision-making in clinical treatment and follow-up for patients with RAML.

In the past decades, SAE had been gradually used to treat RAML (7, 11). It was reported that the treatment response of SAE was satisfactory with a 27% shrinkage rate in 10 years of follow-up (20), but there was still a proportion of patients who exhibited a negative response (**Supplementary Figure 3**). Recently, there had been some studies investigating the factors influencing the response to SAE, while they were confined to small-scale cases and lacked individually quantitative evaluation indicators. Meanwhile, based on high-throughput radiological features, radiomics had been extensively utilized in prognostic models for solid tumors in recent years (21). However, radiomics had not yet been applied to the field of SAE in RAML, and its current main application lay in distinguishing renal masses, particularly in differentiating renal fat-poor angiomyolipoma and renal cell carcinoma (22). Therefore, it was necessary to formulate a robust model that integrated radiomics and clinical features to predict the effectiveness of initial SAE in RAML.

Superselective catheterization ensured precise embolization of arteries supplying tumors, thereby enabling minimally invasive interventions to preserve renal function and minimize complications (23, 24). Systematic reviews had demonstrated a 6.9% complication rate following SAE with an average follow-up period of 39 months, which was notably lower compared to the 12% complication rate observed after partial nephrectomy with a median follow-up period of 8 years (25).

The volume shrinkage in the cohort was significant and within the reported range of 27% to 55.1% (11, 20, 26). In the clinical model, tumor shrinkage rate was significantly related to angiomyogenic tissue ratio, which was consistent with previous studies (27, 28). Hocquelet et al. (9) reported a significant difference in tumor volume reduction between RAMLs with less than 50% fat and those with more than 50% fat (84% vs. 50%; *p* < 0.00001). Victor Prigent et al. (11) also thought that a low-fat content predicted greater volume reduction. By the end of the study’s follow-up, the angiomyogenic tissue ratio in the tumor was 40.8% ± 26.2%, which was significantly smaller than the pretreatment ratio of 59.8% ± 27.6% (*p* < 0.0001). Thus, it could be inferred that it was the angiomyogenic tissue that affected the embolization effect and could be significantly shrunk by SAE. It might be due to the relatively abundant blood supply of the angiomyogenic tissue, leading to more pronounced ischemic response following embolization. To better represent the tumor components responding to SAE, the study directly used angiomyogenic tissue ratio as the risk factor instead of the fat component. Furthermore, it had been reported that the volume reduction ratio of RAMLs varied with the post-SAE follow-up time. Patatas et al. (29) showed that the majority of RAML shrinkage occurred within the first year following embolization and Lee et al. (30) found that the greatest reduction occurred in the early years after embolization before gradually plateauing. Inoue et al. (31) reported that the reduction rates of RAMLs were approximately 55% (3–12 months) vs. 66% (1–3 years). Despite variations in embolization agents and strategies employed in the above studies, the volume reduction following prophylactic embolization gradually increased as post-SAE time extended. Greater volume reduction was also associated with the presence of aneurysms. In the study, aneurysms tended to appear in “fat-poor” AMLs rather than “fat-rich” AMLs. The dysmorphic and immature vascular tissue present in RAMLs was prone to the formation and rupture of aneurysms (32). With the enlargement of the tumor, immature vascular components might induce intra-tumoral aneurysms and resulted in rupture (28). Whether in terms of improving embolization efficacy or preventing rupture, aneurysm was an important factor that required serious attention.

In recent years, radiomics has been used for the diagnosis, treatment response, and survival prediction of renal tumors (33, 34). Radiomics analysis offers objective image information and is applied to identify subtle changes beyond visual assessment on radiological images. Previous investigations had shown that CT texture analysis could be used for differentiating AML without visible fat (AML.wovf) from renal cell carcinoma (RCC). Nie et al. (22) developed a radiomics nomogram to differentiate 36 AMLs.wovf from 84 clear cell renal cell carcinomas (ccRCCs) based on CMP and NP CT images, resulting in an AUC of 0.896. Jian et al. (33) integrated texture features and urine creatine from 19 AMLs and 50 RCCs, and they found that MRI texture analysis could quantitatively discriminate these renal tumors with an AUC of 0.931. However, the application of radiomics in RAML primarily focuses on distinguishing renal tumors, with extremely limited use in evaluating the efficacy of SAE.

Integrating radiomics into predictive studies involves a multi-step process, including feature extraction, selection, and classification, aiming to reduce overfitting and construct robust predictive models (35). However, there is no universal approach, as the performance of machine learning methods varies based on the application and/or data type (36). In the study, we selected three machine learning algorithms, namely, logistic regression, SVM, and DT, to build the radiomics model. The result showed that the radiomics model built by logistic regression had the best performance, but the difference between logistic regression and the other methods was not statistically significant.

There are certain limitations in this study. Firstly, it had a retrospective single-center design, which may introduce selection bias. Secondly, the sample size was limited for a radiomics research. To avoid overfitting, we performed fivefold cross-validation. Finally, the study lacked radiomics features extracted from the corticomedullary phase and plain CT. It was expected to build a radiomics model based on multi-phase scans to mine tumor features more comprehensively and improve the performance of the model.

In conclusion, the study developed a combined radiomics-clinical nomogram for predicting the positive response to initial SAE in RAML. It could serve as an effective complementary tool for traditional imaging to predict the SAE efficacy quantitatively and individually before intervention.

## Data availability statement

The raw data supporting the conclusions of this article will be made available by the authors, without undue reservation.

## Ethics statement

The studies involving humans were approved by Biomedical Research Ethics Committee of Peking University First Hospital. The studies were conducted in accordance with the local legislation and institutional requirements. The ethics committee/institutional review board waived the requirement of written informed consent for participation from the participants or the participants’ legal guardians/next of kin because The data are anonymous. This is a retrospective study.

## Author contributions

LZ: Data curation, Methodology, Writing – original draft, Writing – review & editing. LTS: Writing – review & editing. LTT: Writing – review & editing. CS: Writing – review & editing. YH: Writing – review & editing. YZ: Writing – review & editing. GH: Writing – review & editing. FZ: Writing – review & editing. ZY: Supervision, Writing – review & editing. WJ: Supervision, Writing – review & editing.
