# Supplementary material for: The radiomics-clinical nomogram for predicting the response to initial superselective arterial embolization in renal angiomyolipoma, a preliminary study
Source: Front Oncol. 2024 Mar 5;14:1334706. doi: 10.3389/fonc.2024.1334706 (PMC10949893; doi:10.3389/fonc.2024.1334706)
Supplement: Supplementary file 1 [file DataSheet_1.zip › Supplementary Material/Supplementary_Material.docx]

Supplementary Material


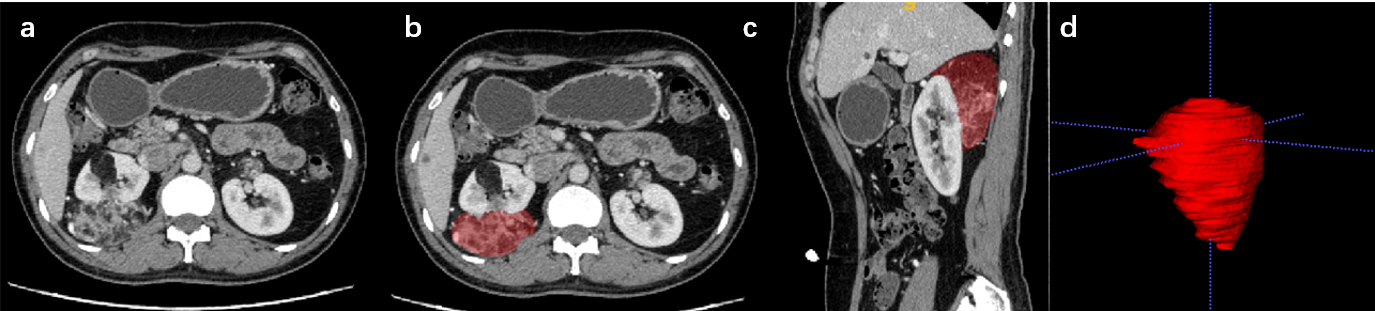


Fig.S1 Case of a 47-year-old female with asymptomatic AML of the right kidney. (A) The axial unenhanced CT image shows visible macroscopic fat on right kidney. (B) Manual tumor segmentation on the axial slice by ITK-SNAP software. (C) Tumor segmentation displays on the sagittal slice. (D) Three-dimensional volumetric reconstruction.


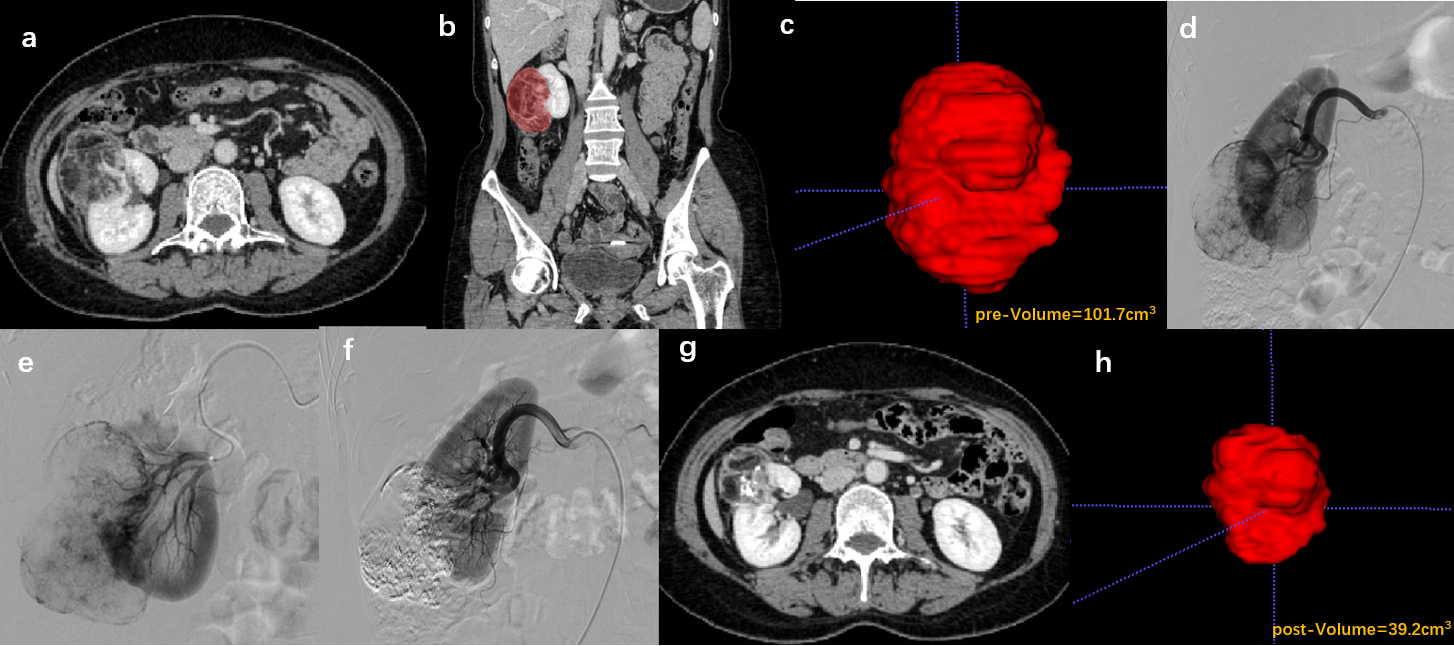


Fig.S2 Case of a 59-year-old female with asymptomatic AML of the right kidney. (A-C) The Preprocedural CT image and manual tumor segmentation on the 3D reconstruction (pre-V=101.7cm3). (D, E) Pre-embolization angiography shows feeding arteries and tumor stain. (F) After superselective embolization with lipiodol and PVA, postembolization DSA scan shows no residual stain. (G, H) The postprocedural CT 3 months later shows lipiodol distribution within the RAML and the reduction of enhanced tissue. The tumor shrinkage rate is 61.5% (post-V=39.2cm3).


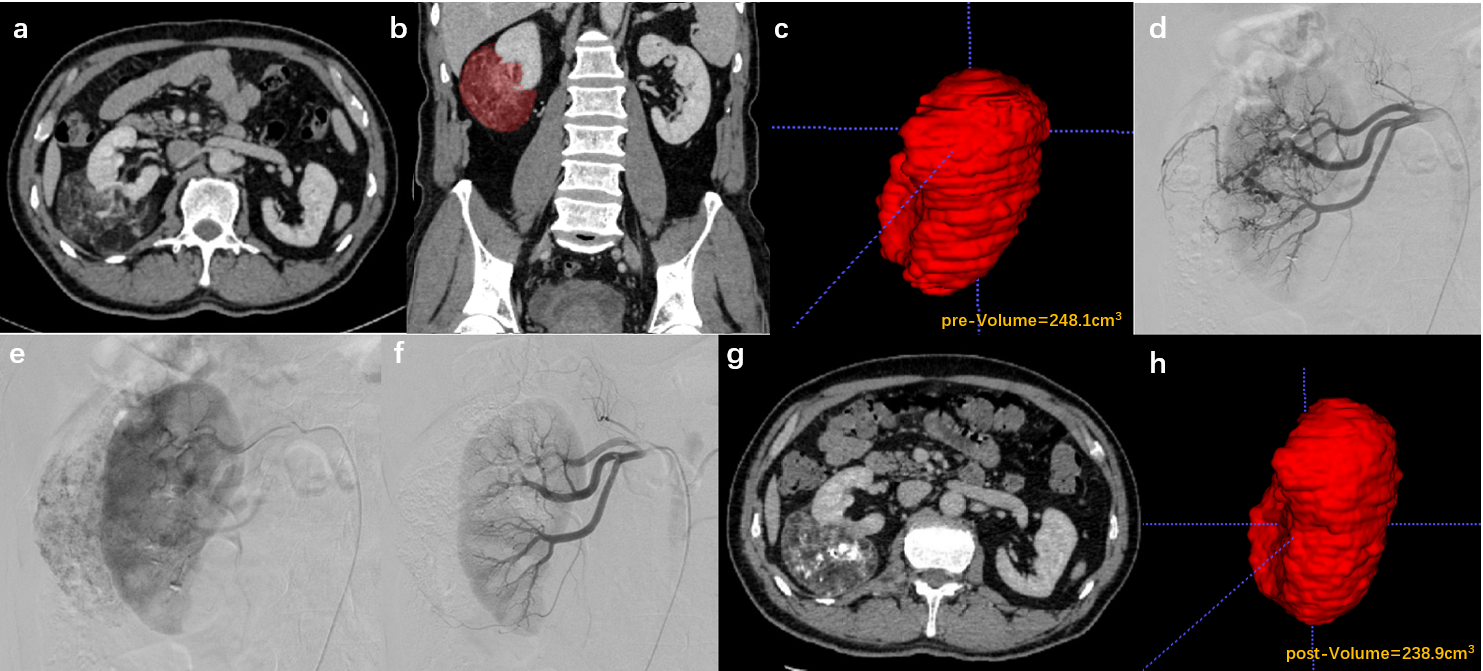


Fig.S3 A 65-year-old patient hospitalized for right chronic flank pain. (A-C) The Preprocedural CT image and manual tumor segmentation on the 3D reconstruction (pre-V=248.1cm3). (D, E) Pre-embolization angiography shows feeding arteries, aneurysms and tumor stain. (F) After superselective embolization of three arterial branches with lipiodol and PVA, the final angiographic scan shows no residual stain. (G, H) The postprocedural CT 3 months later shows lipiodol distribution within the RAML and the tumor shrinkage rate is just 3% (post-V=238.9cm3).

Table S1. Code of in-house software written by python.

| Software | Code |
| --- | --- |
| Python | def segThRatio(img, mask, th = -20):  img_arr = sitk.GetArrayFromImage(img)  mask_arr = sitk.GetArrayFromImage(mask)  volume = np.sum(mask_arr)  mask_region = np.zeros(img_arr.shape)  mask_region[img_arr > th] = 0  mask_region[img_arr <= th] = 1  fat_ratio = np.sum(mask_region * mask_arr) / volume  mask_region = 1 - mask_region  meanCT = np.mean(mask_region * mask_arr * img_arr) |

Table S2. Selected features of the radiomics model

| Index | Radiomics features |
| --- | --- |
| 1 | firstorder.Median |
| 2 | firstorder.RootMeanSquared |
| 3 | firstorder.Skewness |
| 4 | gldm.DependenceVariance |
| 5 | gldm.LowGrayLevelEmphasis |
| 6 | gldm.SmallDependenceLowGrayLevelEmphasis |
| 7 | glrlm.LongRunHighGrayLevelEmphasis |
| 8 | glrlm.ShortRunLowGrayLevelEmphasis |
| 9 | glszm.GrayLevelVariance |
| 10 | glszm.ZoneEntropy |
| 11 | ngtdm.Coarseness |
| 12 | shape.SurfaceVolumeRatio |

Table S3. Spearman correlation coefficient between different factors.

|  | Rad-score | Aneurysm | Post-SAE time | Angiomyogenic tissue | Maximum diameter |
| --- | --- | --- | --- | --- | --- |
| Rad-score | 1 |  |  |  |  |
| Aneurysm | 0.0648 | 1 |  |  |  |
| Post-SAE time | 0.0083 | 0.1277 | 1 |  |  |
| Angiomyogenic tissue | 0.7245 | 0.1829 | -0.0721 | 1 |  |
| Maximum diameter | -0.6004 | 0.3826 | 0.1108 | -0.3798 | 1 |

Table S4. Delong test for the different models

| Model | P value |
| --- | --- |
| Radiomics-Clinical | 0.303 |
| Radiomics-Comb | 0.053 |
| Clinical-Comb | 0.726 |

Radiomics, radiomics model; Clinical, clinical model; Comb, radiomics-clinical model.
