# Supplementary figures and images for: The radiomics-clinical nomogram for predicting the response to initial superselective arterial embolization in renal angiomyolipoma, a preliminary study
Source: Front Oncol. 2024 Mar 5;14:1334706. doi: 10.3389/fonc.2024.1334706 (PMC10949893; doi:10.3389/fonc.2024.1334706)

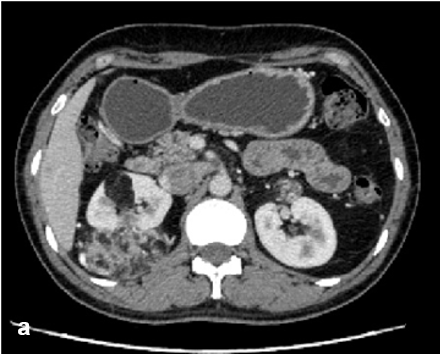

Supplement: Supplementary file 1 [file DataSheet_1.zip › Supplementary Material/Fig S1A.tif]

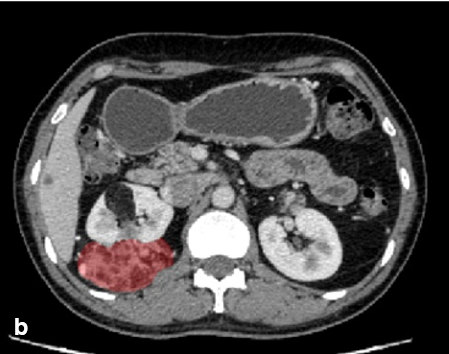

Supplement: Supplementary file 1 [file DataSheet_1.zip › Supplementary Material/Fig S1B.tif]

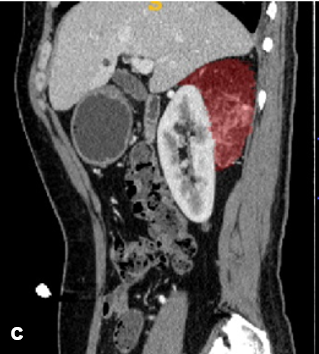

Supplement: Supplementary file 1 [file DataSheet_1.zip › Supplementary Material/Fig S1C.tif]

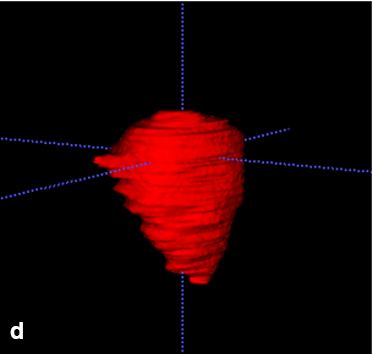

Supplement: Supplementary file 1 [file DataSheet_1.zip › Supplementary Material/Fig S1D.tif]

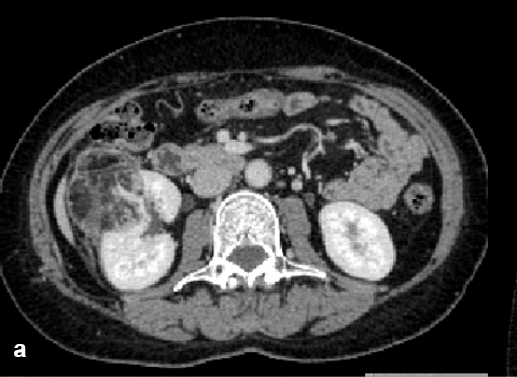

Supplement: Supplementary file 1 [file DataSheet_1.zip › Supplementary Material/Fig S2A.tif]

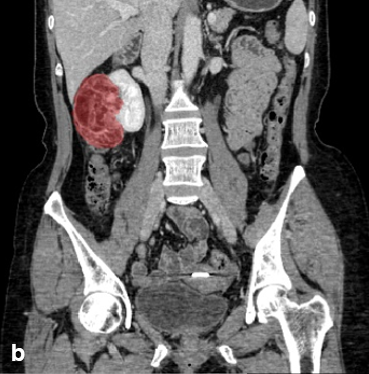

Supplement: Supplementary file 1 [file DataSheet_1.zip › Supplementary Material/Fig S2B.tif]

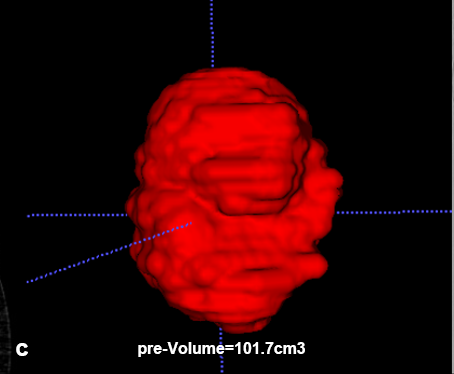

Supplement: Supplementary file 1 [file DataSheet_1.zip › Supplementary Material/Fig S2C.tif]

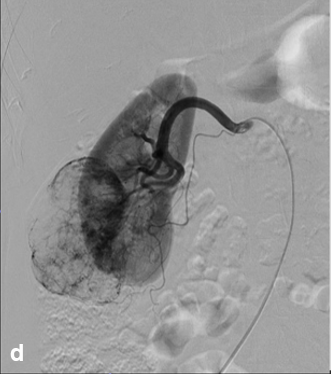

Supplement: Supplementary file 1 [file DataSheet_1.zip › Supplementary Material/Fig S2D.tif]

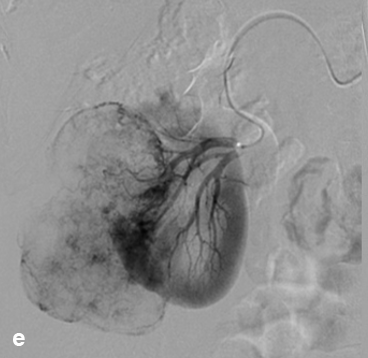

Supplement: Supplementary file 1 [file DataSheet_1.zip › Supplementary Material/Fig S2E.tif]

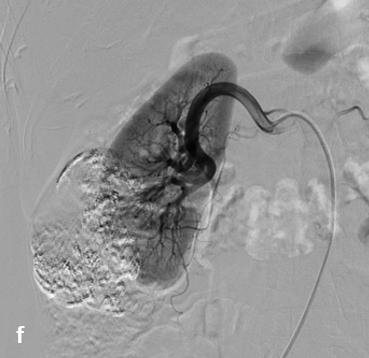

Supplement: Supplementary file 1 [file DataSheet_1.zip › Supplementary Material/Fig S2F.tif]

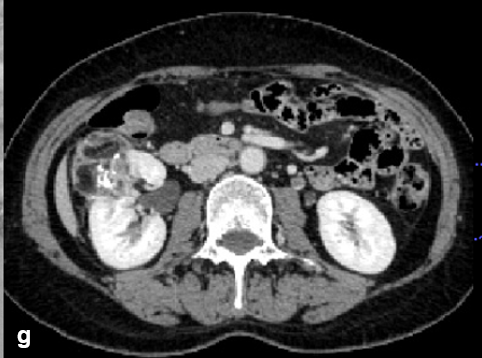

Supplement: Supplementary file 1 [file DataSheet_1.zip › Supplementary Material/Fig S2G.tif]

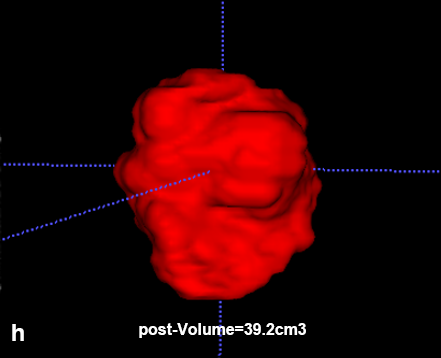

Supplement: Supplementary file 1 [file DataSheet_1.zip › Supplementary Material/Fig S2H.tif]

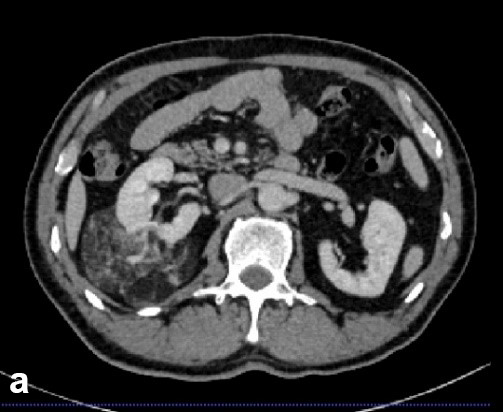

Supplement: Supplementary file 1 [file DataSheet_1.zip › Supplementary Material/Fig S3A.tif]

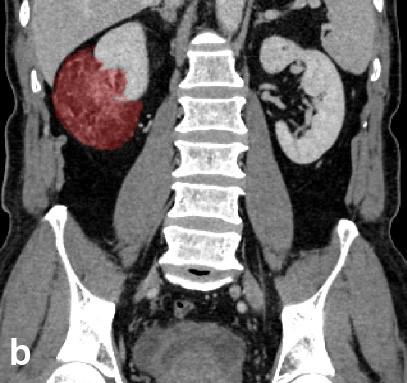

Supplement: Supplementary file 1 [file DataSheet_1.zip › Supplementary Material/Fig S3B.tif]

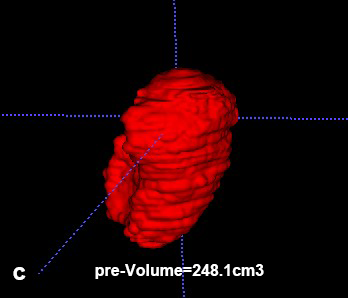

Supplement: Supplementary file 1 [file DataSheet_1.zip › Supplementary Material/Fig S3C.tif]

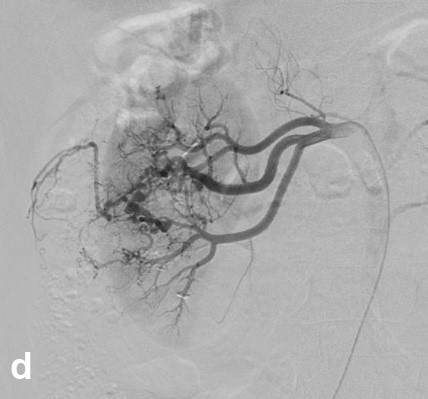

Supplement: Supplementary file 1 [file DataSheet_1.zip › Supplementary Material/Fig S3D.tif]

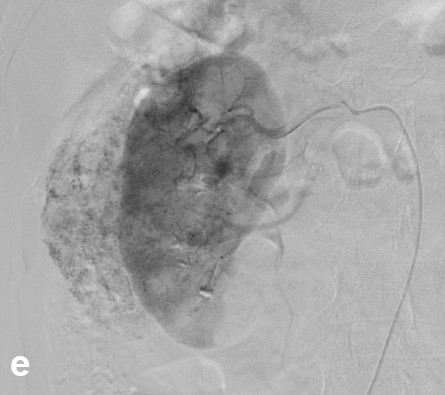

Supplement: Supplementary file 1 [file DataSheet_1.zip › Supplementary Material/Fig S3E.tif]

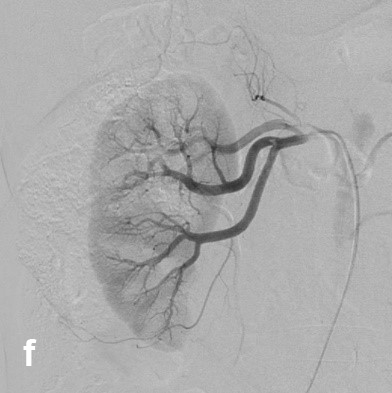

Supplement: Supplementary file 1 [file DataSheet_1.zip › Supplementary Material/Fig S3F.tif]

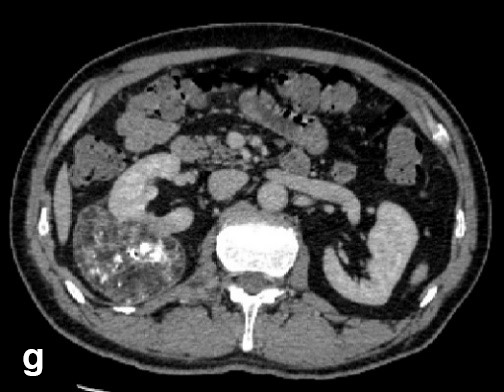

Supplement: Supplementary file 1 [file DataSheet_1.zip › Supplementary Material/Fig S3G.tif]

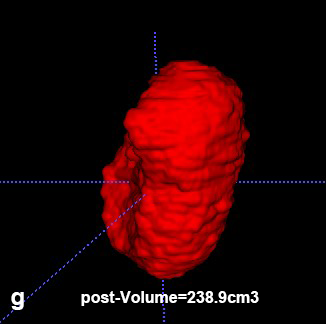

Supplement: Supplementary file 1 [file DataSheet_1.zip › Supplementary Material/Fig S3H.tif]
